# Characterization of bacteriophage vB_KleM_KB2 possessing high control ability to pathogenic *Klebsiella pneumoniae*

**DOI:** 10.1038/s41598-023-37065-5

**Published:** 2023-06-17

**Authors:** Qin Peng, Zimeng Ma, Qing Han, Fangfang Xiang, Lushuang Wang, Yibin Zhang, Yuting Zhao, Jianing Li, Yaxin Xian, Yihui Yuan

**Affiliations:** 1 Ministry of Education Key Laboratory for Ecology of Tropical Islands, College of Life Sciences, Hainan Normal University, Haikou, 571158 China; 2 State Key Laboratory of Marine Resource Utilization in South China Sea, Hainan University, Haikou, 570228 China

**Keywords:** Biotechnology, Genetics, Microbiology

## Abstract

*Klebsiella pneumoniae* is a widespread pathogen of several human diseases. The emergence of multidrug-resistant *K. pneumoniae* makes the treatment of these diseases a significant challenge. The application of bacteriophages is a potential approach for dealing with the emergence of multidrug-resistant pathogenic bacteria. This study isolates a novel bacteriophage vB_KleM_KB2 that infects the multidrug-resistant clinical isolates of *K. pneumoniae*. The bacteriophage exhibits a short latent period of 10 min, and can effectively lyse the bacterium within 60 min. Notably, the bacteriophage can completely inhibit the growth of the host bacterium at the initial concentration of 10^7^ CFU/mL with a low multiplicity of infection of 0.001, which proves its high lytic activity. Furthermore, the bacteriophage shows high environmental tolerances, which can facilitate the practical application of the bacteriophage. Analysis of the bacteriophage genome shows that the bacteriophage possesses a novel genome sequence and can represent a new bacteriophage genus. Considering the high lytic activity, short latent period, high stability, and novel genetic background, bacteriophage vB_KleM_KB2 enriches the bacteriophage library and provides a new alternative for controlling the diseases caused by multidrug-resistant pathogenic *K. pneumoniae*.

## Introduction

Antibiotic resistance has become one of the most extensive public health threats in the world. Tremendous multidrug-resistant (MDR), extensively drug-resistant (XDR), and even pandrug-resistant (PDR) pathogenic bacteria appear, which are difficult or almost impossible to be treated^1^. *Klebsiella pneumoniae* is a widely existing gram-negative pathogenic bacterium that belongs to the *Enterobacteriaceae* family. It colonizes the gastrointestinal tract, skin, and nasopharynx of humans or other mammals to cause the diseases of urinary tract infection, abdominal infection, meningitis, suppurative liver abscess, septicemia, nosocomial and community-acquired pneumonia^2–4^. Attributing to the application of antibiotics during hospital treatment, the *K. pneumoniae* strains are more likely to become the dominant bacteria in the pharyngeal flora, which is because that *K. pneumoniae* is not inherently resistant to most antibiotics, but works as a notorious “collector” of multidrug-resistant plasmids^3^. Thus, pneumonia infection caused by *K. pneumoniae* accounted for a higher proportion among the nosocomial acquired pneumonia infection^5^. Recently, lots of *K. pneumoniae* strains have been found to have resistance against β-lactams, aminoglycosides, quinolones, and some other antibiotics^6^. Such properties make *K. pneumoniae* one of the “ESKAPE” pathogens, including pathogens of *Enterococcus faecium, Staphylococcus aureus, K. pneumoniae, Acinetobacter baumannii, Pseudomonas aeruginosa,* and *Enterobacter* spp., referencing the capacity to escape the biocidal activity of antibiotics through multiple resistance mechanisms and lead to the most common and severe medical related multidrug-resistant infections^7^. Among them, due to the resistance of the last four pathogenic species to carbapenem and cephalosporin, they are listed as priorities by the World Health Organization for research and development of new antibiotics^8^. Therefore, it’s urgent to discover novel agents for controlling the pathogenic *K. pneumoniae*.

Bacteriophage (Phage) is a kind of virus that can infect and kill bacteria, which makes it a potential alternative for controlling antibiotic-resistant pathogenic bacteria. Compared with antibiotic therapy, phage therapy shows several advantages, including high host-bacteria specificity, self-limiting properties, high efficiency at low multiplicity of infection (MOI), relatively simple process and low cost for preparation, and high activity in inhibiting the formation of biofilm^9^. Furthermore, phages are considered to be the most abundant and diverse living organisms on the earth, whose numbers usually exceed their bacterial hosts, which provides an almost unlimited resource for exploring new phage isolates^10,11^. Various environments, including oceans, freshwater, high-salt environments, soils, deserts, polar regions, or other organisms, are found to be habitats of the phage^12,13^. Because of the need to explore new agents for controlling antibiotic-resistant *K. pneumoniae* and the potential of phage in killing bacteria, many *K. pneumoniae* phages have been isolated from various environments on the earth, including wastewater, seawater, and human intestines^14^. The potential application of these phages in controlling pathogenic *K. pneumoniae* has also been explored. Among them, some *K. pneumoniae* phages show high potential in treating *K. pneumoniae*-mediated lobar pneumonia in mice^15,16^. The phage KpJH46Φ2 is found to successfully treat the limb-threatening prosthetic knee *K. pneumonia* infection by intravenous injection^17^. Phages that target the multidrug-resistant *K. pneumoniae* have also been isolated^2^. These findings all prove the high possibility of the phage in treating the disease caused by *K. pneumoniae*.

However, the same as antibiotic resistance, to escape phage killing, the host bacteria can evolve to obtain phage resistance^18^. Benefiting from the high diversity of phage, phage therapy can be used as a kind of customized and personalized treatment method, and the application of a “phage cocktail” would be a promising strategy for avoiding the generation of phage resistance^19^. As for the phage isolates, large burst size, short latent period, and broad host range would be desirable features for the construction of a “phage cocktail”^20^. It is generally believed that the use of lytic phages with a broad host range is more conducive to combating bacterial infections than the use of phages with a narrow host range. Compared to antibiotics, even phages that are considered to have a broad host range usually show a narrower spectrum of activity^21^. By mixing phages with different genetic backgrounds that target pathogenic bacteria with different potential phage resistance to construct a “phage cocktail”, the generation of phage resistance can be significantly avoided, and the treatment effect of phage therapy would be increased^15,22^. Therefore, the establishment of a mature phage library with a vast reserve of phage isolates is vital for the clinical application of phage therapy^23^.

In this study, a phage named vB_KleM_KB2 was isolated, which can be classified as a new phage genus, and its potential for controlling the multidrug-resistant pathogenic *K. pneumoniae* has been evaluated*.* The phage showed the advantages of high lysis activity, short latent period, and high storage stability, which can facilitate the practical application of this phage for treating diseases caused by *K. pneumoniae*. Furthermore, the genomic analysis of the phage proves that the phage possesses a novel genetic background, indicating that the phage represents a novel phage genus. The isolation of this phage enriches the phage library and provides a promising candidate for constructing a “phage cocktail” targeting multidrug-resistant pathogenic *K. pneumoniae*.

## Results

### Electron microscopy observation of phage virions

**Figure 1:**
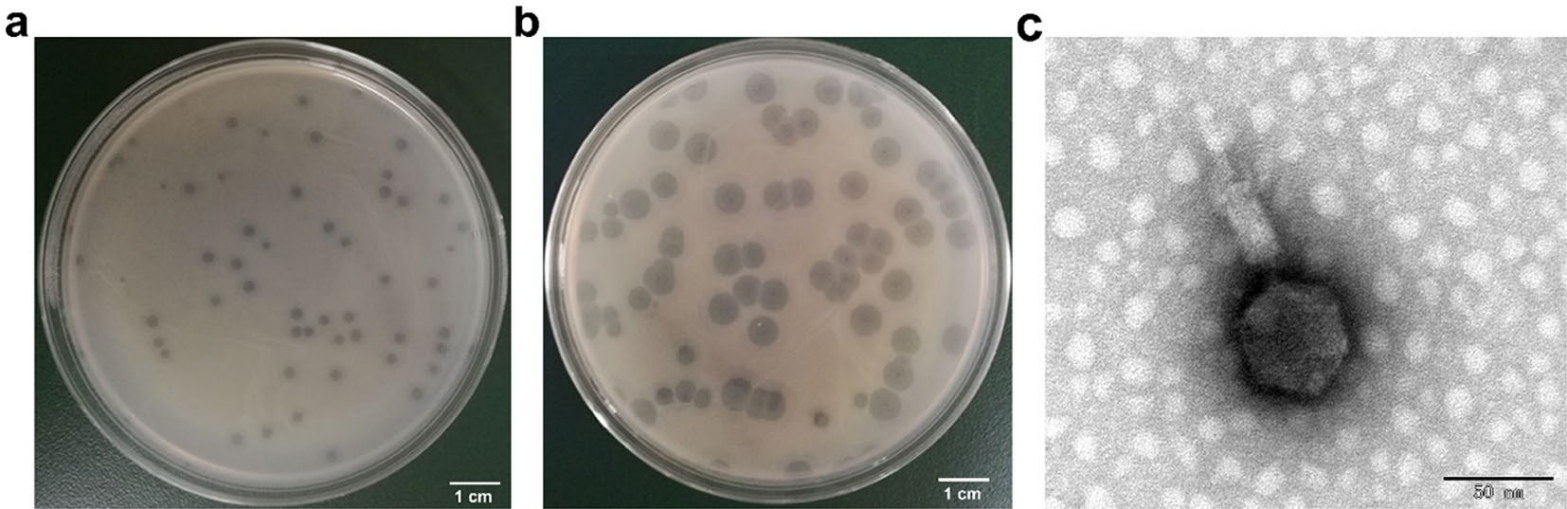
Morphological observation of phage vB_KleM_KB2. Plaques formed by phage vB_KleM_KB2 on the lawn of bacterial strain 0915 after cultivating for 16 h (**a**) and 48 h (**b**). (**c**) Virion morphology of phage vB_KleM_KB2 observed by TEM.

Using the sewage collected from the Diankun River in Haidian island (Haikou, Hainan, China), one phage, named vB_KleM_KB2, was isolated (CCTCC No. M 20221832). This phage forms a plaque with a clear center with a diameter of 1 mm and a turbid rim with a diameter of 4 mm on the bacterial lawn of *K. pneumoniae* strain 0915 after cultivating for 16 h (Fig. 1a). After cultivating for an additional 32 h, the diameter of the turbid rim reaches 10 mm, while the diameter of the clear center remains 1 mm (Fig. 1b). The transmission electron microscopy (TEM) observation of the phage particle shows that phage vB_KleM_KB2 possesses a 50-nm diameter isometric head, a 31-nm-long tail needle, and a 37-nm-long sheath and 17-nm-wide baseplate (Fig. 1c). Based on the morphology of the phage particle, the phage vB_KleM_KB2 is classified as a member of class Caudoviricetes within the family Myoviridae.

### Host range of phage vB_KleM_KB2

Among the 15 bacterial strains used for testing the phage host range, only *K. pneumoniae* strains 2106 and 0915, but not the other strains, can be infected by phage vB_KleM_KB2. These two sensitive *K. pneumoniae* strains are β-lactams-resistant and carbapenems-resistant and belong to multidrug-resistant clinical isolates^2^. Although the other two strains of the *K. pneumoniae* species have also been used for determining the phage host range, they cannot be infected by the phage (Table 1). These results indicate that the phage vB_KleM_KB2 maintains high bacterial specificity, which can avoid the influence on normal bacterial flora during practical application in the human body.

**Table 1:** Host range of the *Klebsiella* phage vB_KleM_KB2 phage.

Strains	Species	Phage sensitivity^a^	Strain resource
2106	*Klebsiella pneumoniae*	+	A clinical isolate^2^
0915	*Klebsiella pneumoniae*	+	A clinical isolate^2^
1025	*Klebsiella pneumoniae*	−	A clinical isolate^2^
2404	*Klebsiella pneumoniae*	−	A clinical isolate^2^
40396	*Escherichia coli*	−	A clinical isolate^24^
40309	*Escherichia coli*	−	A clinical isolate^24^
40482	*Escherichia coli*	−	A clinical isolate^24^
3AB	*Acinetobacter baumannii*	−	A clinical isolate
2AB	*Acinetobacter baumannii*	−	A clinical isolate
4AB	*Acinetobacter baumannii*	−	A clinical isolate
6AB	*Acinetobacter baumannii*	−	A clinical isolate
7AB	*Acinetobacter baumannii*	−	A clinical isolate
YP III	*Yersinia pseudotuberculosis*	−	^25^
Sau01	*Staphylococcus aureus*	−	A clinical isolate
GR−8	*Bacillus pumillus*	−	^26^

^a^+, sensitivity;  −, non-sensitivity.

### one-step growth curve of phage vB_KleM_KB2

**Figure 2:**
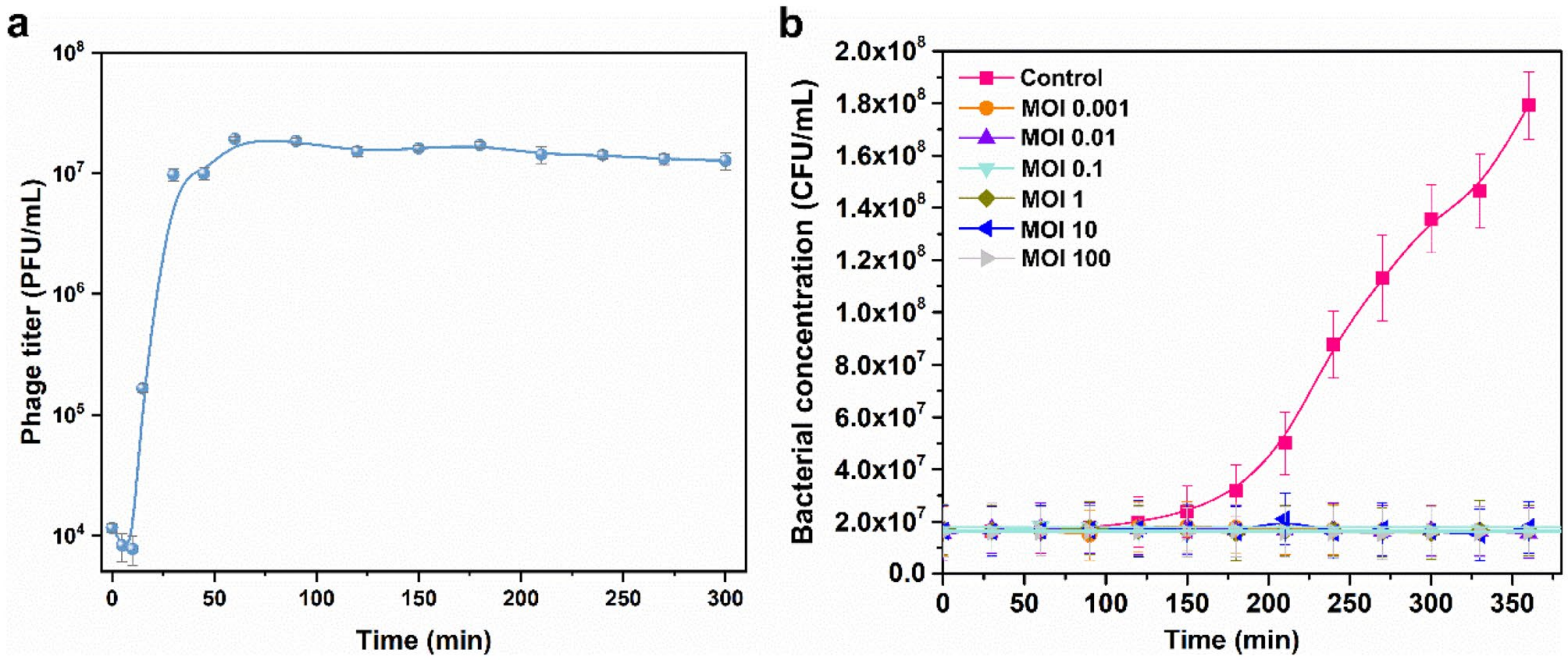
Effect of phage on host bacterial growth. (**a**) One-step growth curve of phage vB_KleM_KB2. (**b**) Effects of the multiplicity of infection on bacterial growth. The strains were treated with the phage of different MOIs, and the treatment without phage was used as a control. For each treatment, the test was performed by three replicates, and the mean value and the standard deviation of each datum were shown.

The latent time and the burst size of the phage are critical for the practical application of the phage. The analysis of the one-step growth curve shows that the phage vB_KleM_KB2 maintains a latent time of about 10 min. A quick rise of the phage titer is observed between 10 and 60 min after the co-cultivation of the phage with the host bacteria, and a plateaus stage is reached after co-cultivating for 60 min with the highest phage concentration up to 10^7^ PFU/mL (Fig. 2a). Based on the phage titer at 60 min, the burse size of this phage is calculated to be approximately 63 PFU/cell.

### Influence of phage MOI on lytic activity

The lytic activity of the phage is another critical factor that determines the potential for application of the phage. The detection of the bacterial concentration shows that the growth of the *K. pneumoniae* strains 0915 with the initial concentration of approximately 10^7^ CFU/mL is completely inhibited by the phage for at least 360 min, proving the high control ability of the phage to the bacterial strain (Fig. 2b). By treating the *K. pneumoniae* strains 0915 with phage of different concentrations, even with the MOI as lower as 0.001, the phage still shows a high inhibition ability to the growth of the bacterium, and the growth of the host strain is almost totally inhibited (Fig. 2b). This result indicates that this phage can be used at an ultralow MOI, which is attributed to the short latent time and the high burst size of the phage. The ultralow effective MOI can reduce the cost for practical application of the phage.

### Stability and environment adaptability of phage vB_KleM_KB2

**Figure 3:**
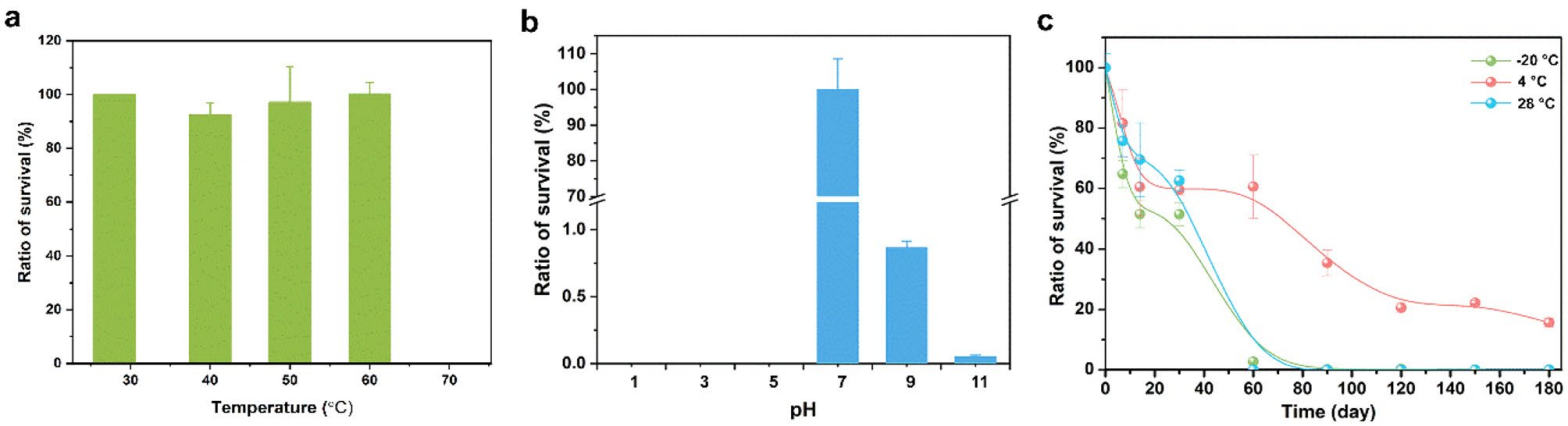
Characteristics of phage vB_KleM_KB2. (**a**) Thermal tolerance of the phage under different temperatures. (**b**) Acid–base tolerance of the phage under conditions of varying pH values. (**c**) Storage stability of the phage at − 20 °C, 4 °C, and 28 °C, respectively. For each treatment, the test was performed by three replicates, and the mean value and the standard deviation of each datum were shown.

The other features that influence the practical application of the phage are stability and environmental tolerance. The temperature tolerance analysis shows that even with a temperature as high as 60 °C, the phage vB_KleM_KB2 maintains 90% of the initial viability after being treated for 30 min (Fig. 3a). A sharp decrease in phage viability was observed after being treated at 70 °C, and only 0.05% of the phage remained viable after 30 min of treatment. The phage vB_KleM_KB2 only shows a narrow pH tolerance and can only be stable at pH 7, which is close to the pH of human body fluid (Fig. 3b). The storage stability analysis of the phage reveals that the phage maintains high stability under different temperatures (Fig. 3c). After being kept at 4 °C for 180 days, the phage remains at 15% of its initial viability. After being held at − 20 °C and 28 °C for 30 days, more than 50% of the phage remains viable. The relative low stability at − 20 °C might be attributed to the low resistance of the phage to the freezing and thawing process, which might lead to the inactivation of the phage. In consideration of the high efficiency of the phage in lysing the host bacterial strain at an ultralow MOI, the undemanding requirements on the storage condition would reduce the cost for cold chain transportation of the phage during practical application.

### Inhibition effect of phage on the growth of host bacteria under different pH

**Figure 4:**
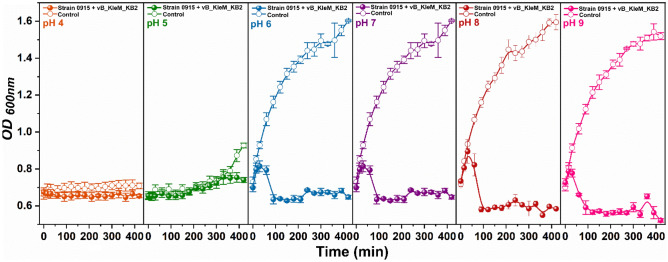
Influence of pH on the inhibition ability of the phage to the growth of the host bacterium. The growth of the bacterium with the treatment of phage was determined at different pH values, and the bacterial culture without phage added was used as a control. The test was carried out with technical triplicates, and the mean value and the standard deviation of each datum were shown.

The pathogenic *K. pneumoniae* is found to localize in different environmental conditions. Thus, the ability of the phage to control pathogens under different conditions is needed for practical application. The influence of pH on the inhibition ability of the phage to the host bacteria has been analyzed. The result shows that, at pH 4 and pH 5, the growth of the host bacteria is inhibited by the environmental pH (Fig. 4). At a pH higher than pH 6, the host bacteria show a normal growth state, and the addition of phage with an MOI of 0.001 can efficiently inhibit the growth of the host bacteria, indicating the phage can be used at a broad pH range from pH 6 to pH 9. Although the phage itself shows low stability by treating with different pH values, the inhibition ability of the phage to the growth of the bacteria is effective at a broad pH, which is because the inhibition on the growth of the bacteria depends on the replication of the phage inside the bacterial cells and is independent from the environmental pH.

### Whole-genome analysis

**Figure 5:**
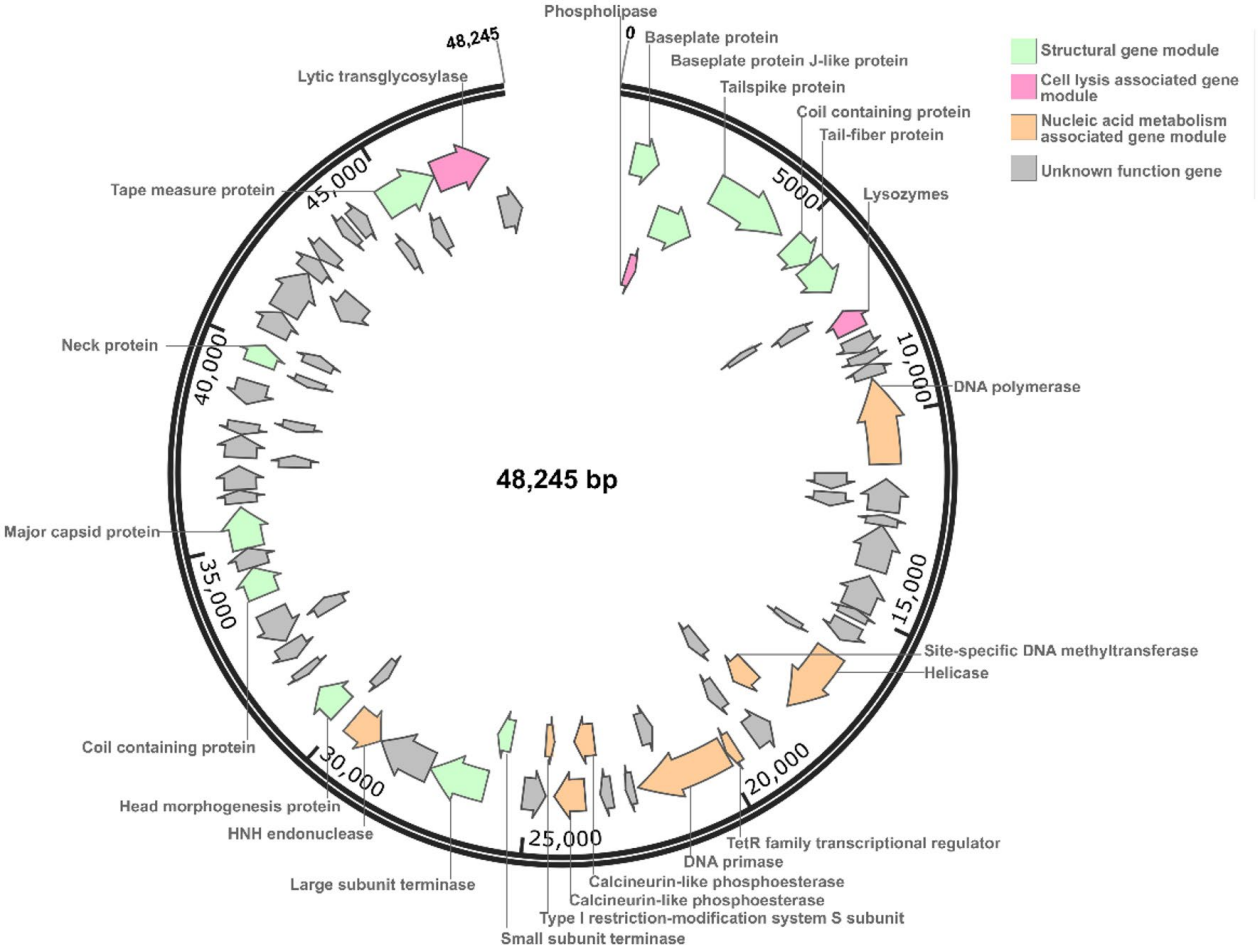
Genome structure of *Klebsiella* phage vB_KleM_KB2. The different gene modules are marked with different colors, including the structural gene module (green color), the cell lysis associated gene module (red violet), and the nucleic acid metabolism associated gene module (orange color). The genes with unknown functions are indicated by gray color.

The complete genome of phage vB_KleM_KB2 is a linear double stranded DNA with a genome size of 48,245 bp and G + C content of 48.89% (Fig. 5). The genome of the phage encodes 71 open reading frames (ORFs), of which 44 are transcribed in a forward direction, 27 are transcribed in a reverse direction, and no tRNA genes and no repeats are found in the genome. Besides, no virulence factor is found, guaranteeing the safety of the phage for practical application in the human body. By comparing these genes with the NR database, 67 genes are found to have significantly matched homologous sequences in the database (E-value ≤ 10^–3^), among which 55 genes show similarity with the genes of *Klebsiella* phages, including 24 functionally annotated genes, and 4 genes are unique to the phage vB_KleM_KB2. Although the functions of numerous genes in the phage genomes are still unclear, it is believed that genes can be preserved only when they are beneficial to the survival of phages^27^. Among them, 12 ORFs are annotated as phage structural proteins, including *gp*1, *gp3-gp*6, *gp*37, *gp*38, *gp*42, *gp*47, *gp*49, *gp*58, and *gp*68. These genes encode the head protein, neck protein, and tail protein of the phage virion, respectively. A total of 9 ORFs are annotated as DNA metabolism associated proteins, and 3 ORFs are annotated as cell lysis associated proteins. Most functional genes of the phage are modular distribution, and the genes with similar functions cluster together.

The genes *gp*9 and *gp*70 are annotated as cell lysis protein-encoding genes, which are predicted to encode lysozymes and lytic transglycosylase, respectively. These genes are distributed at termini of the genome and are highly similar to genes of *K. pneumoniae* phages (> 97% similarity) (Table S1). The gene *gp*70 shows high similarity with the genes from *Klebsiella* phage vB_KpnM_FZ14, 1611E-K2-1, vB_KpnM_KpV52, vB_KpnM_15-38_KLPPOU148, JD001, vB_KpnM_IME346, and MEW1 (Fig. S1). Based on the functions of proteins encoded by genes *gp*9 and *gp*70, both work as phage endolysin, which catalyzes the hydrolysis of the peptidoglycan network structure and damages the physical integrity of bacterial cell walls. However, these two endolysins belong to different functional types. The gene *gp*9 encodes N-acetylmuramidases, and the *gp*70 gene encodes lytic transglycosylase, which all act on the β-1,4-glycosidic bond between N-acetylteichoic acid and N-acetylglucosamine, but generate different hydrolysates^28^. *K. pneumoniae* is a gram-negative bacterium with an outer membrane structure in the outer layer of its cell wall. The gene product of *gp*2 is annotated as phospholipase, which can specifically recognize the phosphatidylglycerol and possesses the function to hydrolyze the phospholipid to damage the bacterial cell membrane^29^. The holin-endolysin lysis system exists in almost all dsDNA phages. At the same time, there is no holin-encoding gene in the genome of phage vB_KleM_KB2, indicating that this phage possesses a new holin-encoding gene or harbors a new strategy for lysing bacterial host.

There are 9 genes in the phage genome that encode nucleic acid metabolism related proteins, including *gp*13, *gp*23-*gp*24, *gp*28, *gp*29, *gp*33-*gp*35, and *gp*40. By searching against the NR database in NCBI, the gene *gp*23 is predicted to encode ATP-dependent DNA helicase, which plays a catalytic role during the replication of the phage genomic DNA. Gp23 shows the highest similarity (96.54% similarity) with the protein from bacterial strain *K. pneumoniae*, but exhibits a distant genetic relationship with the protein from the *K. pneumoniae* phages, including phage vB_KpnM_FZ14, 1611E-K2-1, vB_KpnM_KpV52, vB_KpnM_15-38_KLPPOU148, JD001, vB_KpnM_IME346, MEW1, and vB_KpnM_KpV79, with similarity less than 56% (Fig. S2A). These results indicate that the *gp*23 maybe obtain from the host bacterial genome during the infection process. The gene *gp*24 encodes DNA methyltransferase (MTase), which plays a role in protecting the phage genomes from host encoded restriction-modification (R-M) systems^30^. Both genes *gp*33 and *gp*34 are predicted to encode calcineurin-like phosphoesterase, and the downstream gene *gp*35 is predicted to encode type I restriction-modification system S subunit. Restriction-modification system is reported to provide bacteria with immunity against phage infection, and the S subunit works in recognizing specific DNA sequences^31^. Together with *gp*24, these gene products might give the phage resistance to the restriction-modification system of the host bacteria. The gene product of gene *gp*40 is annotated as HNH endonuclease. What’s interesting is that the N-terminal (1–82 aa) of protein Gp40 shows the highest similarity of 81.93% with protein from *Klebsiella* phage vB_KpnM_IME346, while the C-terminal (79–290 aa) of protein Gp40 shows the highest similarity of 57.73% with that of *P. aeruginosa* (Fig. S2B). The HNH endonuclease is widely distributed in bacteria, archaea, eukaryotes, viruses, or bacteriophages, which plays a vital role in the genome recombination of the phages^32^. The formation of gene *gp*40 might be due to the recombination of the gene from *P. aeruginosa* with a gene from *Klebsiella* phage.

### Comparative genomics analysis of *Klebsiella* phage vB_KleM_KB2

**Figure 6:**
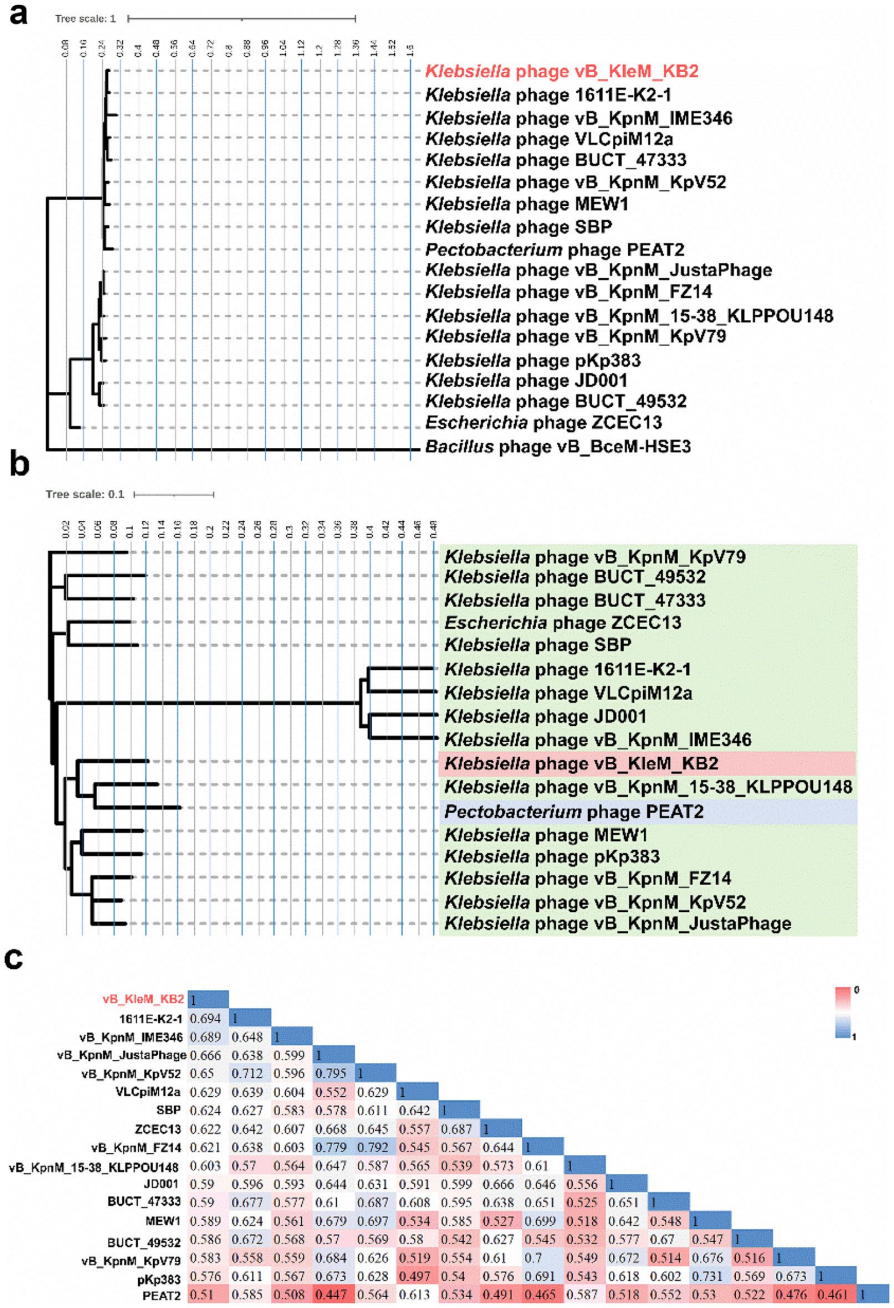
Comparative genomics analysis of *Klebsiella* phage vB_KleM_KB2. (**a**) The phylogenetic tree is constructed based on the DNA polymerase. The phylogenetic tree was generated using the neighbor-joining method and bootstrap analysis (1000 replicates) in MEGA X. (**b**) Phylogenetic tree is constructed based on the whole genomes. The phylogenetic tree was generated using the neighbor-joining method and bootstrap analysis (1000 replicates) in Clustal Omega 1.2.4. The scale bar represents 0.1 substitutions per nucleotide position. (**c**) The similarity matrix of the phage genome was generated by searching against the NCBI database.

By searching the NCBI database, 16 phage genomes are found to show more than 50% similarity with the genome of *Klebsiella* phage vB_KleM_KB2 (Table S2, Fig. S3). Among these phage genomes, three genes are identified as core genes using the software Coregene 5.0 (Table S3). Except for one gene that is predicted to encode a protein with an unknown function, the other two core genes are predicted to encode baseplate protein and DNA primase, respectively. The phylogenetic tree constructed using these DNA polymerases shows that *Klebsiella* phage vB_KleM_KB2 and 1611E-K2-1 clustered in the same main evolutionary branch (Fig. 6a; Table S4). The results of the phylogenetic tree constructed by comparing the whole genome of these 17 phages showed that the phage vB_KleM_KB2 clustered in the same main evolutionary branch and different sub-branches with *Klebsiella* phage SBP and *Escherichia* phage ZCEC13, but it reveals a distant evolutionary relationship (Fig. 6b). Except for *Pectobacterium* phage PEAT2, which belongs to the *Peatvirus* genus, the other 15 phages belong to the *Jedunavirus* genus. As for the whole genome, the genome of phage vB_KleM_KB2 shows the highest similarity with that of phage 1611E-K2-1 (Accession No.: MG197810.1) with 69.4% identical (Fig. 6c). According to the cutoff criteria for genera established by the International Committee on Taxonomy of Viruses (ICTV) Bacterial and Archaeal Viruses Subcommittee, phages are considered to be the same genus if their whole-genome nucleotide sequences exhibit more than 70% identity^33^. Therefore, phage vB_KleM_KB2 could be classified as a new phage genus. The genomes of these phages are analyzed for collinearity and the results show that the genomes of *Klebsiella* phage vB_KleM_KB2 and vB_KpnM_15-38_KLPPOU148, as well as *Pectobacterium* phage PEAT2, exhibit entirely consistent linear arrangement (Fig. S4). The results also show that the *Klebsiella* phages exhibit a general genome rearrangement, indicating the high genome mutation rate of the *Klebsiella* phages.

## Discussion

In this study, a novel phage vB_KleM_KB2 was isolated from the sewage samples. This phage shows high host specificity and only infects two of the tested *K. pneumoniae* strains, which are both multidrug-resistant clinical isolates, indicating that this phage might be used as an alternative for controlling diseases caused by *K. pneumoniae*. Compared with the latent periods of the other *K. pneumoniae* phages, which mainly range from 20 to 70 mins^2,15,16,34–39^, the phage vB_KleM_KB2 exhibits a relatively short latent period of only 10 min. Such a short latent period would benefit the application of the phage by shortening the period for controlling the pathogenic bacteria. This phage also shows an ultralow MOI for controlling the growth of the pathogenic bacteria, and the application of the phage with a low MOI of 0.001 can completely inhibit the growth of the host bacteria. The storage stability is of great importance during the practical application of the phage, as the low storage stability will cause the reduction of control ability of the phage to diseases caused by pathogenic bacteria. It’s important that this phage exhibit good environmental tolerance. *K. pneumoniae* can infect the respiratory tract, intestinal tract, urinary tract, blood, wound, liver, and other parts of the human body^40^, the pH values of which are diverse and range from pH 6.0 to pH 9.0^41,42^. The phage vB_KleM_KB2 shows high lytic activity to the *K. pneumoniae* strain within the pH range from pH 6.0 to pH 9.0. Considering all these features, the phage vB_KleM_KB2 would be a promising candidate for controlling the diseases caused by *K. pneumoniae*.

Another feature that benefits the application of this phage is its novel genetic background. Based on the finding that the nucleotide sequence similarity of the whole genome of phage vB_KleM_KB2 is less than 70% with all available phages, the phage vB_KleM_KB2 can be divided into a new phage genus. Compared with the other available *K. pneumoniae* phages, the phage vB_KleM_KB2 harbors a novel genome sequence with several mutations to facilitate its lytic activity. For example, there are two endolysin encoding genes located at two termini of the phage genome, which is the reason for the short multiplication period and fast lytic speed of the phage as the extra endolysin would synergistically lyse the cell wall of the host bacterium. Furthermore, gene *gp*2, which would express at the early stage after phage infection, is annotated to encode the phospholipase. The gene product Gp2 can act on the outer membrane and inner membrane of the *K. pneumoniae* cell to facilitate the bypass of the endolysin to lyse the bacterial cell wall. This may also be the reason for the rapid and efficient lytic activity of the phage. The novel genome sequence enriches the phage library and provides a candidate for the construction of a “phage cocktail” to avoid the generation of phage resistance.

In summary, a novel phage vB_KleM_KB2 that targets the multidrug-resistant clinical isolates of the *K. pneumoniae* strain has been isolated. The features of the phage vB_KleM_KB2, including high lytic activity, short latent period, high stability, good environmental tolerance, and novel genetic background, make the phage a promising alternative for controlling the disease caused by the multidrug-resistant strains of *K. pneumoniae*.

## Materials and methods

### Isolation and preparation of phage

The clinical isolate of multidrug-resistant *K. pneumoniae* strain 0915 was used as an indicator for isolation of the phage^2^. Sewage samples collected from the Diankun River in Haidian island (Haikou, Hainan, China) were used to isolate the phage that infected *K. pneumoniae* strain 0915. The sewage samples were centrifuged at 12,000×*g* for 10 min at 4 °C to remove the solid impurities. Then the supernatants were filtered through a 0.22-μm pore-size membrane filter to remove the bacterial debris. For each sample, the filtered supernatant was added into exponential growth *K. pneumoniae* strain 0915. After co-cultivating at 37 °C for 8 h, the cultures were centrifuged at 8000×*g* for 30 min, and then filtered through a 0.22-μm pore-size membrane filter. The isolation and purification of the phage were carried out using the double-agar overlay method as previously described^43^.

### Microscopy observation of phage virions

To observe the virion morphology, the phage plaques formed on the agar plate were washed off using an SM buffer (10 mM Tris, 100 mM NaCl, and 10 mM MgSO_4_, pH 7.5), and then filtered through a 0.22-μm pore-size membrane filter. The filtered supernatant was collected, and negative staining was carried out with 2% potassium phosphotungstate (pH 7.2) on the copper grid. The morphology of phage particles was observed by transmission electron microscopy (TEM, JEM-1200EX, JEOL, Japan) under 120 kV accelerating voltage.

### Determination of host range

To determine the host range of the phage, bacterial strains of different species, including the species of *K. pneumoniae, Acinetobacter baumannii, Escherichia coli, Yersinia pseudotuberculosis, Staphylococcus aureus*, and *Bacillus pumillus*, were used (Table 1). The determination of the host range was carried out as previously described^2^.

### One-step growth curve

A one-step growth curve of the phage was performed to determine the latent period and phage burst size. The phage was mixed with the host bacterium at an MOI of 1.0 for 5 min for absorption. Subsequently, the mixture was centrifuged at 10,000×*g* for 1 min to remove the non-absorbed phage. Then, the precipitate was resuspended in 50 mL fresh LB broth, and the phage titers in the culture were determined at an interval of 15 min. The one-step growth curve was generated as previously described^44^.

### Determination of physical stability of the phage

The thermal stability of the phage was determined at 28 °C, 40 °C, 50 °C, 60 °C, and 70 °C. The phage suspension with a concentration of 10^8^ PFU/mL was incubated at different temperatures for 30 min and then cooled slowly to room temperature. The phage stored at 28 ℃, nearing room temperature, was used as a control. The phage was incubated in buffer with pH of 1.0, 3.0, 5.0, 7.0, 9.0, and 11.0, for 30 min at 28 °C, and the initial pH value (pH 7.5) of suspension was used as a control. To determine the suitable temperature for storing the phage suspension, the phage suspension was stored at − 20 °C, 4 °C, and 28 °C, for 6 months. The phage titers were determined at 7, 14, 30, 60, 90, 120, 150, and 180 days, after being stored. The phage titer was determined by the double-agar overlay method, and each treatment was performed by three replicates.

### Effect of phage concentration on lytic activity

To determine the effect of different phage concentrations in lysing the host bacterium, the exponential growth *K. pneumoniae* strain 0915 was used. The phage and the bacterium were mixed with a ratio of 100, 10, 1, 0.1, and 0.01, 0.001, respectively, and left to adsorb for 5 min at 37 °C. After that, the mixture was centrifugated at 10,000×*g* for 1 min to remove non-absorbed phage. The precipitate was resuspended in 50 mL fresh LB broth and cultivated at 37 °C with moderate shaking. The bacterial culture without adding phage was used as a control. The bacterial concentration was measured every 30 min.

### Effect of pH on phage lytic activity

To determine the influence of environmental pH on the lytic activity of the phage, the pH of the freshly prepared LB broth was adjusted to 4.0, 5.0, 6.0, 7.0, 8.0, and 9.0, respectively, by using the solution of HCl or NaOH. The phage was mixed with the exponential growth *K. pneumoniae* strain 0915 with a concentration ratio of 0.001. After adsorbing for 5 min at room temperature, the mixture was centrifugated at 10,000×*g* for 1 min, and the precipitate was resuspended in LB broth of different pH values. The mixture was cultivated at 37 °C with moderate shaking and the optical density (OD_600nm_) of the cultures was measured every 30 min.

### Phage DNA extraction, sequencing, and genomic analysis

As previously reported, the genomic DNA of the isolated phage was extracted with phenol–chloroform after treatment with protease K-sodium dodecyl sulfate (SDS)^45^. The purified phage DNA was sent to sequencing by the Sangon Biotech (Shanghai) Co., Ltd. company with the Illumina HiSeq 2500 sequencer. In total, 22,874,944 reads were obtained and assembled into contigs using the SPAdes-3.5.0 software (Illumina, San Diego, CA, USA). The gaps between the contigs were filled by prime walking. The coding sequences (CDSs) in the genome were predicted by FGENESV0 (Softberry, http://linux1.softberry.com) and visualized using SnapGene-V5.2.4.0. Each predicted gene was searched in NCBI non-redundant protein sequences (NR) and CDD databases by using the basic local alignment search tool (BLAST)^46^. The motifs and functional domain compositions of predicted protein were analyzed by Pfam and HHpred database for functional annotation^47,48^. The coding genes of tRNAs were searched by tRNAscan-SE 2.0 (http://lowelab.ucsc.edu/tRNAscan-SE/index.html)^49^. Tandem Repeat Finder software was used to find the repeats in the genome of phage (https://tandem.bu.edu/trf/trf.basic.submit.html)^50^. Phylogenetic analysis of the functional proteins was performed using MEGA X with the neighbor-joining method and bootstrap analysis (1000 replicates) with the ClustalW alignment^51^. By using the software Coregenes 5.0 (https://coregenes.ngrok.io/)^52^, the core genes of phages with more than 50% similarity were obtained (E-value < 10^–5^). A phylogenetic tree of the whole genome nucleotide sequence was generated using the neighbor-joining method and bootstrap analysis (1000 replicates) in Clustal Omega 1.2.4 (https://www.ebi.ac.uk/Tools/msa/clustalo/)^53^. Software Mauve 2.3.1 was used for multiple sequence alignment of the phage genomes^54^.

## Supplementary Information

Supplementary Information.

## Data Availability

The datasets generated and/or analyzed during the current study are available in the NCBI repository (https://www.ncbi.nlm.nih.gov) [Accession Number to Datasets: MT757392.1].
