# Supplementary material for: Characterization of bacteriophage vB_KleM_KB2 possessing high control ability to pathogenic Klebsiella pneumoniae
Source: Sci Rep. 2023 Jun 17;13:9815. doi: 10.1038/s41598-023-37065-5 (PMC10276810; doi:10.1038/s41598-023-37065-5)
Supplement: Supplementary file 1 — Supplementary Information. [file 41598_2023_37065_MOESM1_ESM.docx]

***Supplementary Materials***

**Table S1.** Genome feature of phage vB_KleM_KB2

| **Gene accession** | **strand** | **Nucleotide position** | | **Length (amino acid）** | **Description** | **Best match** | **NCBI match accession** | **Similarity** | **E-value** |
| --- | --- | --- | --- | --- | --- | --- | --- | --- | --- |
|  |  | **start** | **end** |  |  |  |  |  |  |
| *gp*1 | + | 554 | 1228 | 224 | baseplate protein | *Klebsiella* phage vB_KpnM_IME346 | QBZ68915.1 | 98.21% | 3.00E-162 |
| *gp*2 | + | 1228 | 1575 | 115 | phospholipase | *Klebsiella* phage vB_KpnM_IME346 | QBZ68914.1 | 100.00% | 3.00E-76 |
| *gp*3 | + | 1568 | 2776 | 402 | baseplate protein J-like protein | *Klebsiella* phage vB_KpnM_FZ14 | QCG76488.1 | 96.77% | 0 |
| *gp*4 | + | 2773 | 4779 | 668 | tailspike protein | *Klebsiella* phage 1611E-K2-1 | ATS92567.1 | 93.86% | 0 |
| *gp*5 | + | 5064 | 5777 | 237 | coil containing protein | *Klebsiella* phage vB_KpnM_FZ14 | QCG76489.1 | 93.25% | 4.00E-164 |
| *gp*6 | + | 5781 | 6674 | 297 | tail-fiber protein | *Klebsiella* phage vB_KpnM_IME346 | QBZ68907.1 | 97.31% | 0 |
| *gp*7 | - | 6669 | 6905 | 78 | hypothetical protein | *Klebsiella* phage vB_KpnM_KpV52 | YP_00959757 | 97.44% | 1.00E-48 |
| *gp*8 | - | 6902 | 7231 | 109 | hypothetical protein | *Klebsiella* phage vB_KpnM_KpV52 | YP_009597575.1 | 96.33% | 4.00E-69 |
| *gp*9 | - | 7228 | 7788 | 186 | lysozymes | *Klebsiella* phage vB_KpnM_KpV79 | YP_009615320.1 | 98.39% | 1.00E-133 |
| *gp*10 | + | 7957 | 8367 | 136 | hypothetical protein | *Klebsiella* phage vB_KpnM_KpV79 | YP_009615322.1 | 97.06% | 1.00E-91 |
| *gp*11 | + | 8394 | 8675 | 93 | hypothetical protein | *Klebsiella* phage JD001 | YP_007392879.1 | 79.57% | 1.00E-39 |
| *gp*12 | - | 8692 | 9021 | 109 | hypothetical protein | *Klebsiella* phage vB_KpnM_IME346 | QBZ68900.1 | 94.50% | 1.00E-69 |
| *gp*13 | - | 9062 | 11236 | 724 | DNA polymerase | *Klebsiella* phage 1611E-K2-1 | ATS92564.1 | 97.93% | 0 |
| *gp*14 | + | 11456 | 11923 | 155 | hypothetical protein | *Pectobacterium atrosepticum* phage PEAT2 | YP_009702193.1 | 75.63% | 2.00E-48 |
| *gp*15 | - | 11604 | 12416 | 270 | hypothetical protein | *Klebsiella* phage vB_KpnM_KpV52 | YP_009597584.1 | 97.04% | 0 |
| *gp*16 | + | 12024 | 12437 | 137 | hypothetical protein | No hits |  |  |  |
| *gp*17 | - | 12504 | 12761 | 85 | hypothetical protein | Myoviridae sp. | DAE76754.1 | 100% | 1E-56 |
| *gp*18 | - | 12764 | 13894 | 376 | hypothetical protein | *Klebsiella* phage vB_KpnM_IME346 | QBZ68973.1 | 95.45% | 0 |
| *gp*19 | - | 14067 | 14954 | 295 | hypothetical protein | *Klebsiella* phage vB_KpnM_KpV52 | YP_009597587.1 | 80.49% | 1.00E-153 |
| *gp*20 | - | 14959 | 15201 | 80 | hypothetical protein | *Salmonella* phage FSL SP-076 | YP_008240217.1 | 48.61% | 2.00E-17 |
| *gp*21 | + | 15340 | 15822 | 160 | hypothetical protein | *Klebsiella* phage vB_KpnM_IME346 | QBZ68966.1 | 91.88% | 9.00E-108 |
| *gp*22 | + | 15989 | 16201 | 70 | hypothetical protein | *Klebsiella* phage vB_KpnM_IME346 | QBZ68968.1 | 55.41% | 5.00E-18 |
| *gp*23 | + | 16198 | 17934 | 578 | helicase | *Klebsiella* phage 1611E-K2-1 | ATS92563.1 | 57.35% | 0 |
| *gp*24 | + | 17934 | 18584 | 216 | site-specific DNA methyltransferase | Myoviridae sp. | DAE76733.1 | 100.00% | 3E-65 |
| *gp*25 | - | 18526 | 19113 | 195 | hypothetical protein | *Shewanella* phage SFCi1 | ANO58052.1 | 42.61% | 4.00E-09 |
| *gp*26 | + | 18566 | 19054 | 162 | hypothetical protein | *Klebsiella* phage JD001 | YP_007392868.1 | 82.47% | 9.00E-88 |
| *gp*27 | + | 19102 | 19512 | 136 | hypothetical protein | *Rhizobiales* bacterium | WP_112988338.1 | 61.48% | 9.00E-54 |
| *gp*28 | + | 19575 | 19853 | 92 | TetR family transcriptional regulator | *Klebsiella* phage vB_KpnM_IME346 | QBZ68961.1 | 97.83% | 4.00E-58 |
| *gp*29 | + | 19876 | 22233 | 785 | DNA primase | *Klebsiella* phage vB_KpnM_KpV52 | QCG76500.1 | 97.07% | 0 |
| *gp*30 | - | 21388 | 21828 | 146 | hypothetical protein | No hits |  |  |  |
| *gp*31 | + | 22327 | 22542 | 71 | hypothetical protein | *Klebsiella* phage vB_KpnM_KpV52 | YP_009597597.1 | 100.00% | 8.00E-45 |
| *gp*32 | + | 22888 | 23184 | 98 | hypothetical protein | *Klebsiella* phage vB_KpnM_KpV52 | YP_009597600.1 | 100.00% | 3.00E-65 |
| *gp*33 | + | 23184 | 23777 | 197 | calcineurin-like phosphoesterase | *Klebsiella* phage vB_KpnM_FZ14 | QCG76503.1 | 99.44% | 5.00E-125 |
| *gp*34 | + | 23566 | 24333 | 255 | calcineurin-like phosphoesterase | *Klebsiella* phage vB_KpnM_FZ14 | QCG76503.1 | 92.99% | 2.00E-145 |
| *gp*35 | - | 24364 | 24618 | 84 | type I restriction-modification system S subunit | *Klebsiella* phage JD001 | YP_007392858.1 | 100.00% | 9.00E-32 |
| *gp*36 | - | 24542 | 25111 | 189 | hypothetical protein | *Klebsiella* phage vB_KpnM_KpV52 | YP_009597603.1 | 62.89% | 4.00E-72 |
| *gp*37 | + | 25622 | 26077 | 151 | small subunit terminase | *Klebsiella* phage vB_KpnM_FZ14 | QCG76504.1 | 83.55% | 2.00E-82 |
| *gp*38 | + | 26067 | 27515 | 482 | large subunit terminase | *Klebsiella* phage vB_KpnM_FZ14 | QCG76505.1 | 100.00% | 0 |
| *gp*39 | + | 27518 | 28912 | 464 | hypothetical protein | *Klebsiella* phage vB_KpnM_IME346 | QBZ68950.1 | 96.34% | 0 |
| *gp*40 | - | 28927 | 29802 | 291 | HNH endonuclease | *Pseudomonas aeruginosa* | WP_023875451.1 | 57.73% | 1.00E-81 |
| *gp*41 | - | 29799 | 30128 | 109 | hypothetical protein | *Klebsiella* phage vB_KpnM_KpV52 | YP_009597534.1 | 77.06% | 6.00E-50 |
| *gp*42 | + | 30197 | 30958 | 253 | head morphogenesis protein | *Klebsiella* phage vB_KpnM_FZ14 | QCG76507.1 | 100.00% | 0 |
| *gp*43 | - | 31388 | 31594 | 68 | hypothetical protein | *Klebsiella* phage vB_KpnM_KpV52 | YP_009597540.1 | 100.00% | 1.00E-39 |
| *gp*44 | - | 31895 | 32314 | 139 | hypothetical protein | *Klebsiella* phage vB_KpnM_15-38_KLPPOU148 | QGZ13398.1 | 61.44% | 3.00E-52 |
| *gp*45 | - | 32390 | 33235 | 281 | hypothetical protein | *Klebsiella* phage vB_KpnM_KpV79 | YP_009615325.1 | 35.61% | 4.00E-46 |
| *gp*46 | + | 32502 | 32909 | 135 | hypothetical protein | No hits |  |  |  |
| *gp*47 | + | 33656 | 34402 | 248 | coil containing protein | *Klebsiella* phage vB_KpnM_FZ14 | QCG76509. | 98.39% | 6.00E-172 |
| *gp*48 | + | 34414 | 34902 | 162 | hypothetical protein | *Klebsiella* phage vB_KpnM_IME346 | QBZ68937.1 | 94.44% | 1.00E-99 |
| *gp*49 | + | 34905 | 35939 | 344 | major capsid protein | *Klebsiella* phage vB_KpnM_FZ14 | QCG76510.1 | 95.64% | 0 |
| *gp*50 | + | 36044 | 36361 | 105 | hypothetical protein | *Klebsiella* phage vB_KpnM_KpV52 | YP_009597548.1 | 88.57% | 3.00E-61 |
| *gp*51 | + | 36380 | 36964 | 194 | hypothetical protein | *Klebsiella* phage vB_KpnM_IME346 | QBZ68934.1 | 94.85% | 1.00E-129 |
| *gp*52 | + | 36961 | 37317 | 118 | hypothetical protein | *Klebsiella* phage JD001 | YP_007392839.1 | 99.15% | 3.00E-78 |
| *gp*53 | + | 37178 | 37738 | 186 | hypothetical protein | *Klebsiella* phage vB_KpnM_IME346 | QBZ68932.1 | 92.09% | 4.00E-91 |
| *gp*54 | - | 37755 | 38060 | 101 | hypothetical protein | *Pantoea latae* | WP_081138958.1 | 36.17% | 1.00E-07 |
| *gp*55 | - | 38024 | 38314 | 96 | hypothetical protein | *Serratia fonticola* | WP_074029047.1 | 49.40% | 3.00E-19 |
| *gp*56 | - | 38522 | 39100 | 192 | hypothetical protein | *Klebsiella* phage vB_KpnM_KpV52 | YP_009597552.1 | 100.00% | 3.00E-138 |
| *gp*57 | - | 39406 | 39660 | 84 | hypothetical protein | Myoviridae sp. | DAI64976.1 | 53.18% | 2E-23 |
| *gp*58 | + | 39560 | 40063 | 167 | neck protein | *Klebsiella* phage vB_KpnM_FZ14 | QCG76511.1 | 97.01% | 7.00E-115 |
| *gp*59 | + | 40060 | 40413 | 117 | hypothetical protein | *Klebsiella* phage vB_KpnM_KpV52 | YP_009597554. | 100.00% | 2.00E-80 |
| *gp*60 | + | 40400 | 40981 | 193 | hypothetical protein | *Klebsiella* phage vB_KpnM_15-38_KLPPOU148 | QGZ13393.1 | 98.96% | 2.00E-140 |
| *gp*61 | + | 41022 | 42158 | 378 | hypothetical protein | *Klebsiella* phage vB_KpnM_IME346 | QBZ68925.1 | 98.15% | 0.00% |
| *gp*62 | - | 41643 | 42425 | 260 | hypothetical protein | *Pectobacterium atrosepticum* phage PEAT2 | YP_009702230.1 | 88.08% | 6.00E-166 |
| *gp*63 | + | 42172 | 42588 | 138 | hypothetical protein | *Klebsiella* phage vB_KpnM_IME346 | QBZ68924.1 | 99.28% | 3.00E-93 |
| *gp*64 | - | 42615 | 42992 | 125 | hypothetical protein | Myoviridae sp. | DAE76774.11 | 69.3% | 3E-59 |
| *gp*65 | - | 43433 | 43717 | 94 | hypothetical protein | *Klebsiella* phage vB_KpnM_IME346 | QBZ68921.1 | 98.94% | 3.00E-64 |
| *gp*66 | + | 43837 | 44211 | 124 | hypothetical protein | *Klebsiella* phage JD001 | YP_007392827.1 | 100.00% | 3.00E-85 |
| *gp*67 | + | 44398 | 44694 | 98 | hypothetical protein | *Klebsiella* phage vB_KpnM_IME346 | QBZ68919.1 | 97.96% | 5.00E-66 |
| *gp*68 | + | 44694 | 46163 | 489 | tape measure protein | *Klebsiella* phage 1611E-K2-1 | ATS92553.1 | 96.93% | 0 |
| *gp*69 | - | 45434 | 45850 | 138 | hypothetical protein | No hits |  |  |  |
| *gp*70 | + | 46166 | 47590 | 474 | lytic transglycosylase | *Klebsiella* phage vB_KpnM_FZ14 | ATS92552.1 | 97.89% | 0 |
| *gp*71 | + | 47587 | 48237 | 216 | hypothetical protein | *Klebsiella* phage vB_KpnM_KpV79 | YP_009615309.1 | 100.00% | 2.00E-153 |


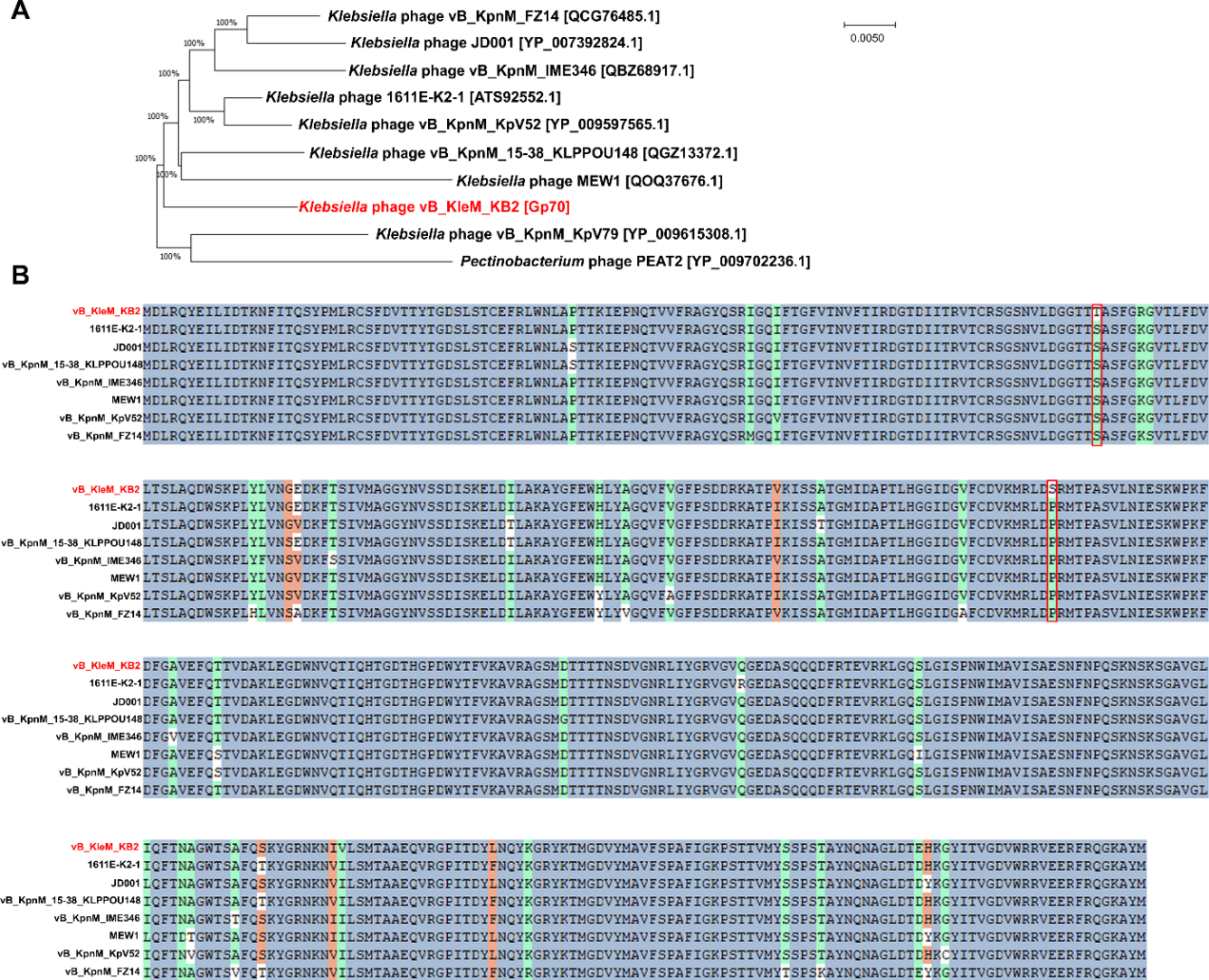


**Figure S1.** Analysis of the functional proteins Gp70 of the *Klebsiella* phage vB_KleM_KB2. (A) Phylogenetic analysis of the lytic transglycosylase. More than 95% similarity with Gp70 was used to construct the phylogenetic tree. The phylogenetic tree was generated using the neighbor-joining method and bootstrap analysis (1000 replicates) in MEGA X. The scale bar represents 0.05 substitutions per nucleotide position. (B) Alignment of the protein Gp70 with the proteins of the *Klebsiella* phage vB_KpnM_FZ14, 1611E-K2-1, vB_KpnM_KpV52, vB_KpnM_15-38_KLPPOU148, JD001, vB_KpnM_IME346, and MEW1.


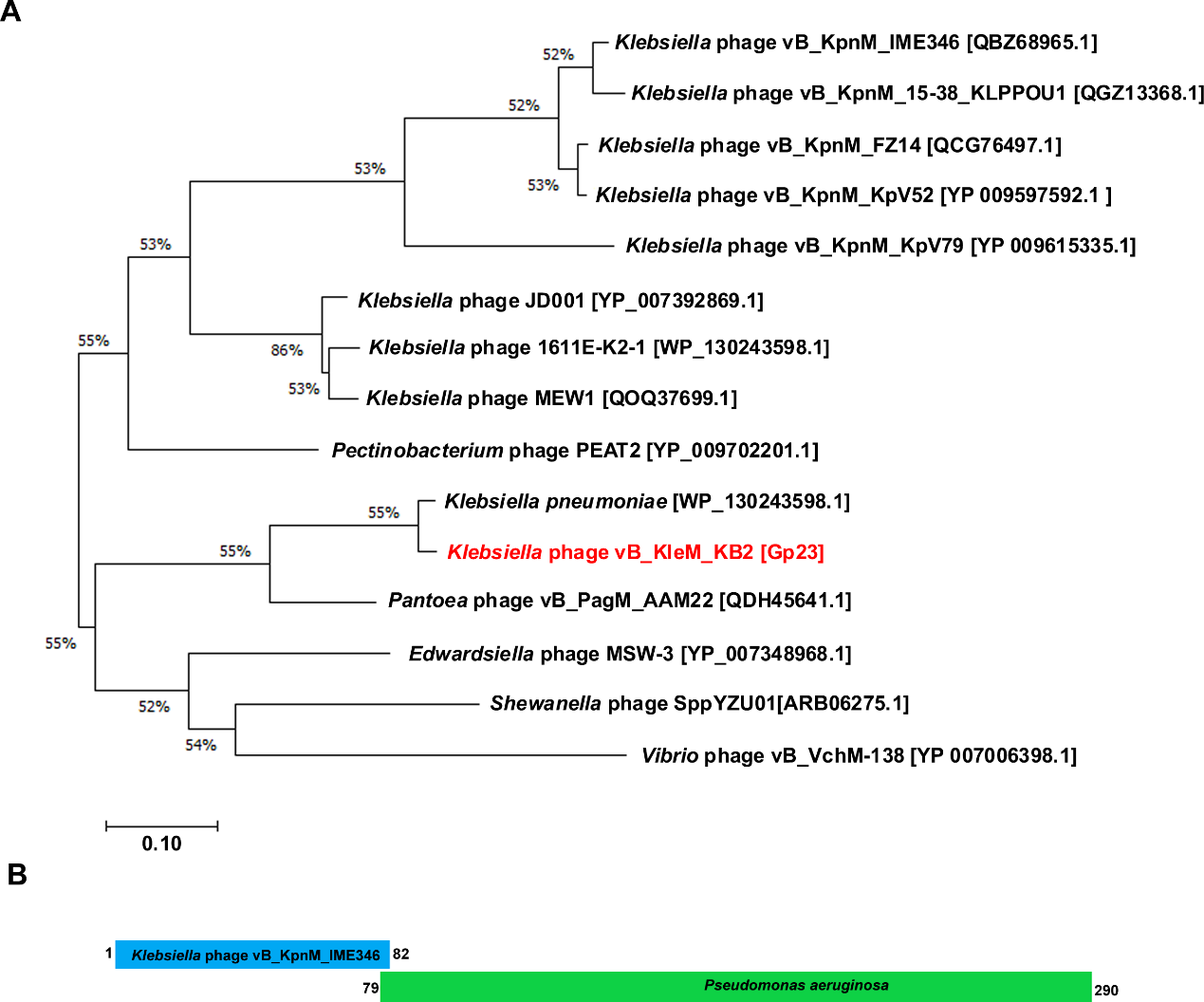


**Figure S2.** Analysis of the functional proteins Gp23 and Gp40 of the *Klebsiella* phage vB_KleM_KB2. (A) Phylogenetic analysis of the helicase. More than 40% similarity with Gp23 was used to construct the phylogenetic tree. The phylogenetic tree was generated using the neighbor-joining method and bootstrap analysis (1000 replicates) in MEGA X. The scale bar represents 0.05 substitutions per nucleotide position. (B) Schematic diagram of the similarity of Gp40.

**Table S2.** The genome information of 17 phages and used for mauve and phylogenetic analysis in the study

| **Genome** | **GenBank Accession** | **Length** | **Type of DNA** | **Isolation source** | **Host** | **Classification** |
| --- | --- | --- | --- | --- | --- | --- |
| *Klebsiella* phage vB_KleM_KB2 | MT757392.1 | 48245 bp | linear DNA | sewage water | *Klebsiella pneumoniae* | Caudoviricete; unclassified |
| *Klebsiella* phage 1611E-K2-1 | MG197810.1 | 47797 bp | linear DNA | - | *Klebsiella pneumoniae* | Caudoviricetes; *Jedunavirus* |
| *Klebsiella* phage vB_KpnM_15-38_KLPPOU148 | MN689778.1 | 49169 bp | linear DNA | - | *Klebsiella pneumoniae* | Caudoviricetes; *Jedunavirus* |
| *Klebsiella* phage vB_KpnM_JustaPhage | OK499978.1 | 48129bp | linear DNA | raw sewage | *Klebsiella pneumoniae* | Caudoviricetes; *Jedunavirus* |
| *Klebsiella* phage vB_KpnM_KpV52 | NC_041900.1 | 47405 bp | linear DNA | sewage | *Klebsiella pneumoniae* | Caudoviricetes; *Jedunavirus* |
| *Klebsiella* phage vB_KpnM_FZ14 | MK521906.1 | 49370 bp | linear DNA | sewage water | *Klebsiella pneumoniae* | Caudoviricetes; *Jedunavirus* |
| *Klebsiella* phage MEW1 | MT894004.1 | 47129 bp | linear DNA | watercourse | *Klebsiella pneumoniae* | Caudoviricetes; *Jedunavirus* |
| *Klebsiella* phage SBP | OP114730.1 | 49371 bp | linear DNA | sewage | *Klebsiella pneumoniae* | Caudoviricetes; *Jedunavirus* |
| *Klebsiella* phage vB_KpnM_IME346 | MK685667.1 | 49482 bp | linear DNA | - | *Klebsiella pneumoniae* | Caudoviricetes; *Jedunavirus* |
| *Klebsiella* phage vB_KpnM_KpV79 | NC_042041.1 | 47760 bp | linear DNA | sewage | *Klebsiella pneumoniae* | Caudoviricetes; *Jedunavirus* |
| *Klebsiella* phage JD001 | JX866719.1 | 48814 bp | linear DNA | hospital | *Klebsiella pneumoniae* | Caudoviricetes; *Jedunavirus* |
| *Klebsiella* phage VLCpiM12a | ON602758.1 | 48833 bp | circular DNA | soil and water | *Klebsiella pneumoniae* | Caudoviricetes; *Jedunavirus* |
| *Klebsiella* phage pKp383 | ON809560.1 | 48837 bp | circular DNA | - | *Klebsiella pneumoniae* | Caudoviricetes; *Jedunavirus* |
| *Klebsiella* phage BUCT_47333 | MZ398021.1 | 47333 bp | linear DNA | sewage | *Klebsiella pneumoniae* | Caudoviricetes; *Jedunavirus* |
| *Klebsiella* phage BUCT_49532 | MZ374361.1 | 49532 bp | linear DNA | sewage | *Klebsiella pneumoniae* | Caudoviricetes; *Jedunavirus* |
| *Escherichia* phage ZCEC13 | ON086804.1 | 48021 bp | linear DNA | - | *Escherichia* spp. | Caudoviricetes; *Jedunavirus* |
| *Pectobacterium* phage PEAT2 | NC_044940.1 | 48659 bp | linear DNA | - | *Pectobacterium* spp. | Caudoviricetes; *Peatvirus* |

**Table S3.** The core genes of the *Klebsiella* phage vB_KleM_KB2, 1611E-K2-1,vB_KpnM_15-38_KLPPOU148, vB_KpnM_JustaPhage, vB_KpnM_KpV52, vB_KpnM_FZ14, MEW1, SBP, vB_KpnM_IME346, vB_KpnM_KpV79, JD001, VLCpiM12a, pKp383, BUCT_47333, BUCT_49532, *Escherichia* phage ZCEC13, and *Pectinobacterium* phage PEAT2

| **Phage** | **Core genes** | | |
| --- | --- | --- | --- |
| *Klebsiella* phage vB_KleM_KB2 | QNI20483.1 | QNI20510.1 | QNI20517.1 |
| *Klebsiella* phage 1611E-K2-1 | ATS92568.1 | ATS92562.1 | ATS92559.1 |
| *Klebsiella* phage vB_KpnM_15-38_KLPPOU148 | QGZ13387.1 | QGZ13366.1 | QGZ13373.1 |
| *Klebsiella* phage vB_KpnM_JustaPhage | UGO49377.1 | UGO49410.1 | UGO49340.1 |
| *Klebsiella* phage vB_KpnM_KpV52 | YP_009597567.1 | YP_009597596.1 | YP_009597531.1 |
| *Klebsiella* phage vB_KpnM_FZ14 | QCG76486.1 | QCG76500.1 | QCG76506.1 |
| *Klebsiella* phage MEW1 | QOQ37678.1 | QOQ37703.1 | QOQ37712.1 |
| *Klebsiella* phage SBP | UYE94765.1 | UYE94791.1 | UYE94803.1 |
| *Klebsiella* phage vB_KpnM_IME346 | QBZ68915.1 | QBZ68960.1 | QBZ68950.1 |
| *Klebsiella* phage vB_KpnM_KpV79 | YP_009615310.1 | YP_009615339.1 | YP_009615274.1 |
| *Klebsiella* phage JD001 | AFZ77623.1 | AFZ77598.1 | AFZ77587.1 |
| *Klebsiella* phage VLCpiM12a | UVX31545.1 | UVX31514.1 | UVX31578.1 |
| *Klebsiella* phage pKp383 | UVD41502.1 | UVD41529.1 | UVD41539.1 |
| *Klebsiella* phage BUCT_47333 | QXG78630.1 | QXG78661.1 | QXG78599.1 |
| *Klebsiella* phage BUCT_49532 | QWY14551.1 | QWY14582.1 | QWY14514.1 |
| *Escherichia* phage ZCEC13 | UPU16065.1 | UPU16112.1 | UPU16093.1 |
| *Pectinobacterium* phage PEAT2 | YP_009702238.1 | YP_009702203.1 | YP_009702215.1 |

**Table S4.** The DNA polymerases of the *Klebsiella* phage vB_KleM_KB2, 1611E-K2-1,vB_KpnM_15-38_KLPPOU148, vB_KpnM_JustaPhage, vB_KpnM_KpV52, vB_KpnM_FZ14, MEW1, SBP, vB_KpnM_IME346, vB_KpnM_KpV79, JD001, VLCpiM12a, pKp383, BUCT_47333, BUCT_49532, *Escherichia* phage ZCEC13, *Pectinobacterium* phage PEAT2 and *Bacillus* phage vB_BceM-HSE3

| **Phage** | **Protein accession** |
| --- | --- |
| *Klebsiella* phage vB_KleM_KB2 | QNI20495.1 |
| *Klebsiella* phage 1611E-K2-1 | ATS92564.1 |
| *Klebsiella* phage vB_KpnM_15-38_KLPPOU148 | QGZ13369.1 |
| *Klebsiella* phage vB_KpnM_JustaPhage | UGO49393.1 |
| *Klebsiella* phage vB_KpnM_KpV52 | AOZ65397.1 |
| *Klebsiella* phage vB_KpnM_FZ14 | QCG76494.1 |
| *Klebsiella* phage MEW1 | QOQ37692.1 |
| *Klebsiella* phage SBP | YP_010684517.1 |
| *Klebsiella* phage vB_KpnM_IME346 | MK685667.1 |
| *Klebsiella* phage vB_KpnM_KpV79 | YP_009615326.1 |
| *Klebsiella* phage JD001 | AFZ77609.1 |
| *Klebsiella* phage VLCpiM12a | UVX31531.1 |
| *Klebsiella* phage pKp383 | UVD41517.1 |
| *Klebsiella* phage BUCT_47333 | QXG78644.1 |
| *Klebsiella* phage BUCT_49532 | YP_010683708.1 |
| *Escherichia* phage ZCEC13 | UPU16153.1 |
| *Pectinobacterium* phage PEAT2 | ATV25067.1 |
| *Bacillus* phage vB_BceM-HSE3 | AWD93085.1 |


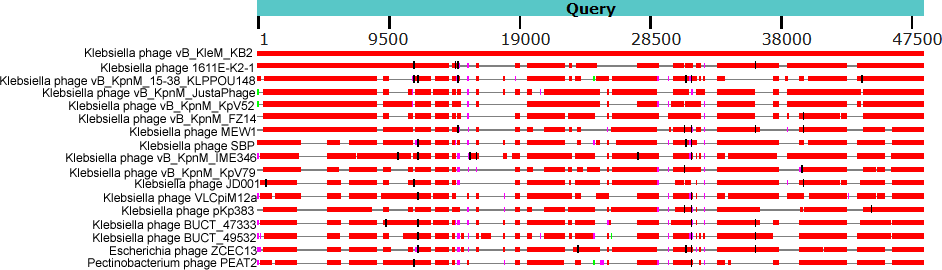


**Figure S3**. Comparison of the whole genome of *Klebsiella* phage vB_KleM_KB2 with the other phages. Only the results of the phage with more than 50% genome similarity are shown. The analysis was performed by searching against the NCBI database by using BLASTn.


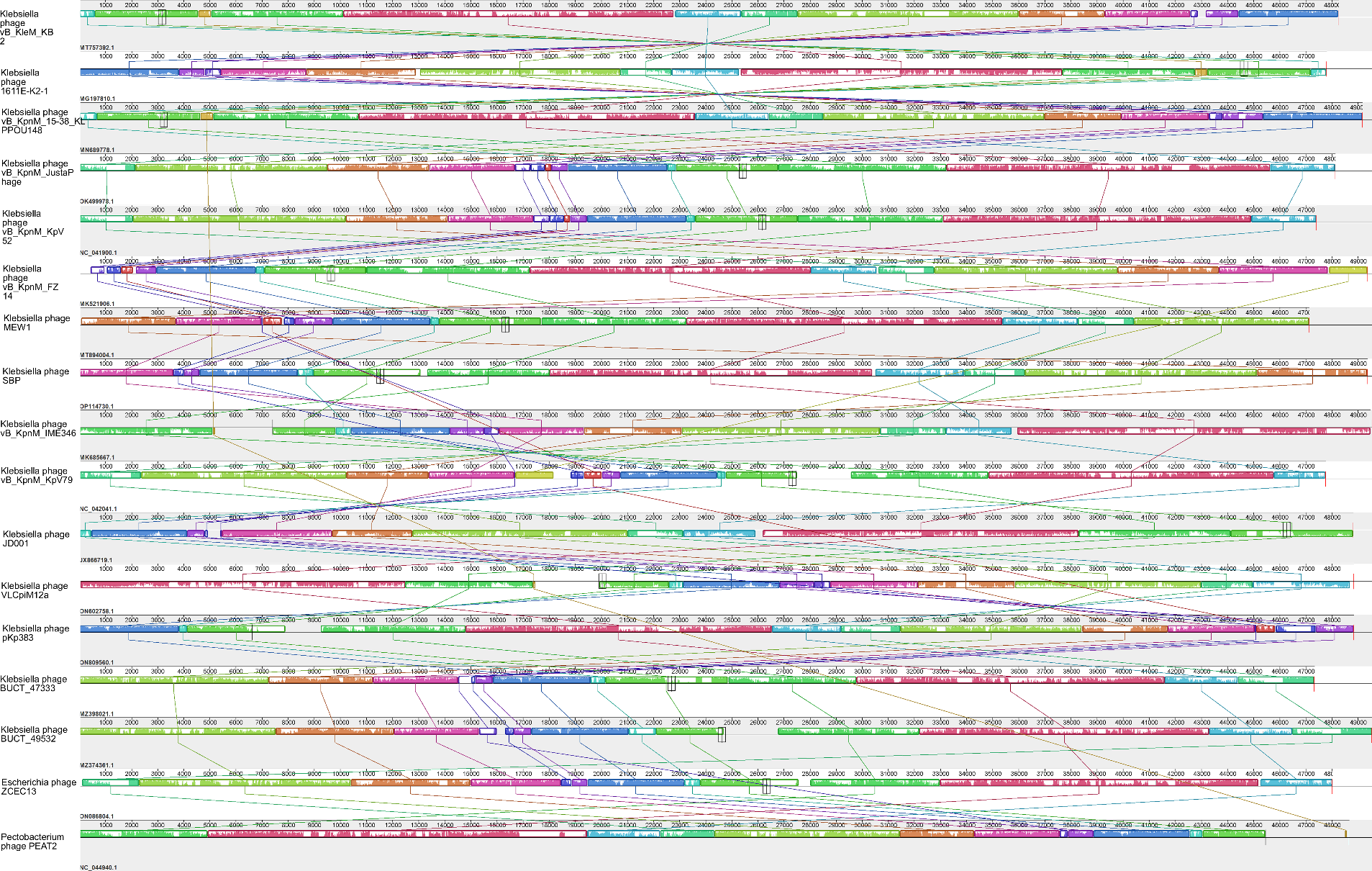


**Figure S4.** Multiple sequence alignment of phage genomes by software Mauve. Genome sequences that show more than 50% similar to phage vB_KleM_KB2 were used for comparison.
